# Lessons on malaria control in the ethnic minority regions in Northern Myanmar along the China border, 2007–2014

**DOI:** 10.1186/s40249-016-0191-0

**Published:** 2016-10-06

**Authors:** Ru-Bo Wang, Jia-Qiang Dong, Zhi-Gui Xia, Tao Cai, Qing-Feng Zhang, Yao Zhang, Yang-Hui Tian, Xiao-Ying Sun, Guang-Yun Zhang, Qing-Pu Li, Xiao-Yu Xu, Jia-Yin Li, Jun Zhang

**Affiliations:** 1 National Institute of Parasitic Diseases, Chinese Center for Disease Control and Prevention, WHO Collaborative Center forTropical Diseases, Key Laboratory of Parasite and Vector Biology, Ministry of Health, Shanghai, 200025 China; 2 Yunnan Representative Office, Health Poverty Action (UK), Kunming, 650020 China

**Keywords:** Malaria control, Ethnic minority regions, Northern Myanmar

## Abstract

**Background:**

For many countries where malaria is endemic, the burden of malaria is high in border regions. In ethnic minority areas along the Myanmar-China border, residents have poor access to medical care for diagnosis and treatment, and there have been many malaria outbreaks in such areas. Since 2007, with the support of the Global Fund to Fight AIDS, Tuberculosis and Malaria (GFATM), a malaria control project was introduced to reduce the malaria burden in several ethnic minority regions.

**Methods:**

A malaria control network was established during the period from 2007 to 2014. Multiple malaria interventions, including diagnosis, treatment, distribution of LLINs and health education, were conducted to improve the accessibility and quality of malaria control services for local residents. Annual cross-sectional surveys were conducted to evaluate intervention coverage and indicators of malaria transmission.

**Results:**

In ethnic minority regions where a malaria control network was established, both the annual malaria incidence (19.1 per thousand per year, in 2009; 8.7, in 2014) and malaria prevalence (13.6 % in 2008; 0.43 % in 2014) decreased dramatically during the past 5–6 years. A total of 851 393 febrile patients were detected, 202 598 malaria cases (including confirmed cases and suspected cases) were treated, and 759 574 LLINs were delivered to populations at risk. Of households in 2012, 73.9 % had at least one ITNs/LLINs (vs. 28.3 %, in 2008), and 50.7 % of children less than 5 years and 50.3 % of pregnant women slept under LLINs the night prior to their visit. Additionally, malaria knowledge was improved in 68.4 % of residents.

**Conclusion:**

There has been great success in improving malaria control in these regions from 2007 to 2014. Malaria burdens have decreased, especially in KOK and WA. The continued maintenance of sustainable malaria control networks in these regions may be a long-term process, due to regional conflicts and the lack of funds, technology, and health workers. Furthermore, information and scientific support from the international community should be offered to these ethnic minority regions to uphold recent achievements.

**Electronic supplementary material:**

The online version of this article (doi:10.1186/s40249-016-0191-0) contains supplementary material, which is available to authorized users.

## Multilingual abstract

Please see Additional file 1 for translations of the abstract into the five official working languages of the United Nations.

## Background

Malaria morbidity and mortality have been reduced in the past decade at the global level. Despite this overall decrease, there were still 198 million confirmed cases and 584 000 deaths reported in 2013 [1]. The malaria burden in border areas is higher than that in certain countries of the Great Mekong Sub-region, such as Thailand, and the actual malaria morbidity and mortality in these regions is likely underestimated [2]. Malaria is one of the most prevalent infectious diseases in Myanmar, especially in the remote area along the border, near Thailand, India, Bangladesh and China [3–5]. Although malaria control along the Thai-Myanmar border is of international concern [6–8], ethnic minority regions in northern Myanmar close to the Chinese border have been a ‘blind spot’ for malaria control.

Myanmar shares a border with China for more than 2 000 km. This border area of Myanmar is primarily composed of several marginalized ethnic minority groups, where heavily forested terrain, poor accessibility to infrastructure, constant cross-border movement of people, and political conflict pose significant challenges to its inhabitants. The lack of health products, funds and health workers results in poor access to malaria diagnosis and treatment for ethnic minority groups in these areas, and malaria outbreaks often occur [9, 10]. In 2003, a malaria outbreak took place in KOK (Kokang Special Region, Shan State), resulting in more than 100 fatalities. The border allows for the unrestricted crossing of people and vehicles, facilitating malaria transmission to neighbouring areas. The transmission of malaria from these ethnic minority regions is one of the largest obstacles for malaria elimination in China [11, 12].

In July 2007, under the support of the Global Fund to Fight AIDS, Tuberculosis and Malaria (GFATM), a malaria control project was introduced to reduce the malaria burden in several ethnic minority regions. The project covered four regions, including KSR2 (Special Region 2^nd^, Kachin State), KOK (Kokang Special Region, Shan State), WA (Special Region 2^nd^, Shan State) and SR4 (Special Region 4^th^, Shan State). Since 2012, the project was expanded to KSR1 (Special Region 1^st^, Kachin State), the fifth region covered by the GFATM project. The project was implemented by an international non-governmental organization (Health Poverty Action, HPA) that had providing public health services in the Northern-Myanmar border area for 20 years. In the present paper, the 7-year impact of this GFATM malaria control project was analysed from 2007 to 2014.

## Methods

### Study location

**Fig. 1:**
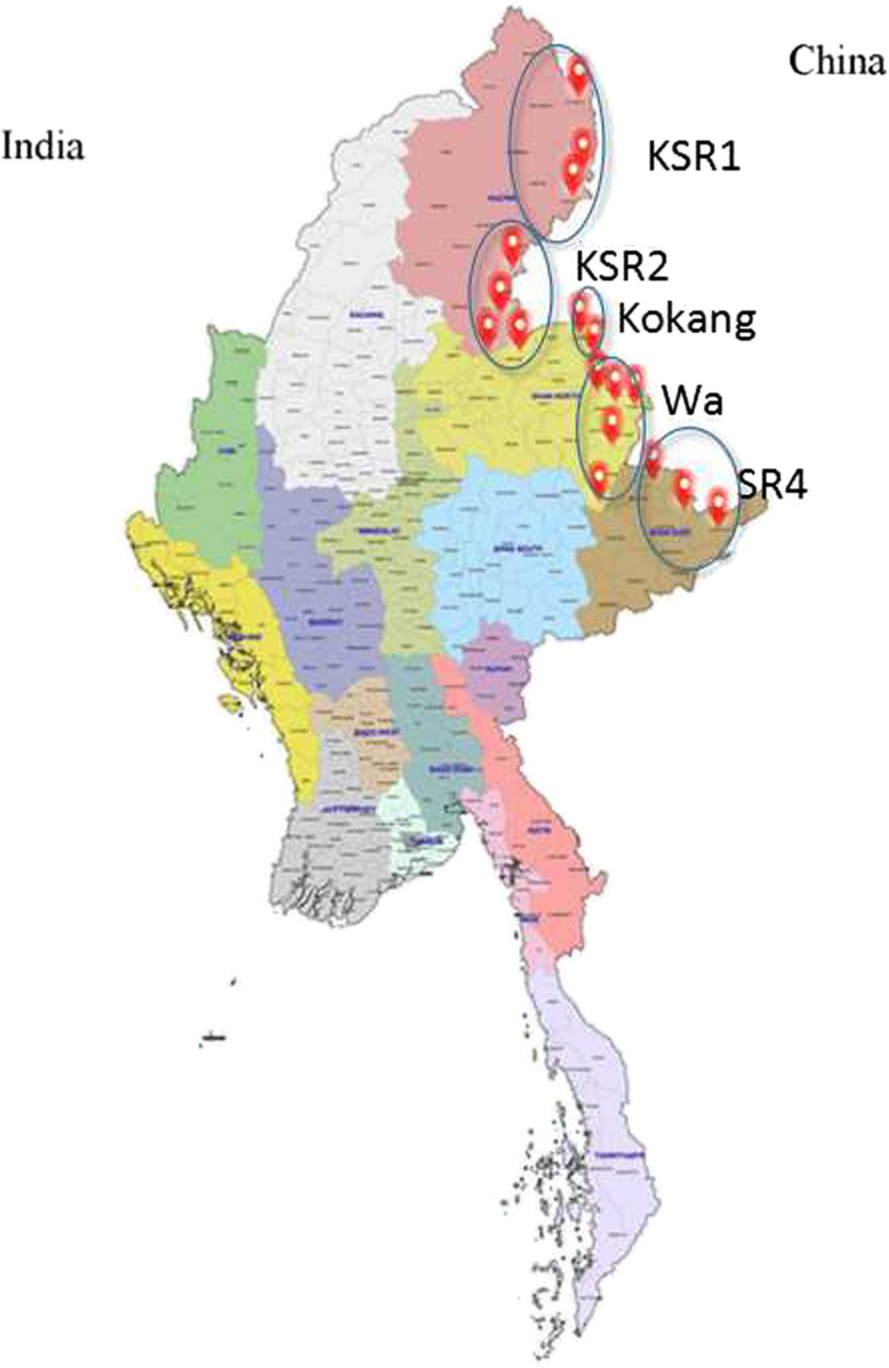
The map of Program area in 2012 (The program townships were marked with *red* hot)

The areas covered by the GFATM project included 14 townships of four regions during 2007–2011 and 17 townships of five regions during 2012–2014 (Fig. 1). There were approximately 546 000 local residents, as estimated by the local government, in five regions. Like much of the surrounding terrain, the areas were mountainous and forested. The local residents were mainly ethnic minorities, such as Kachin, Wa, Kokang, Dai, Lahu, Bulang and Hani, and mobile populations mainly came from China. The towns suffered from poor economies, and the government malaria funds were scant in these five ethnic minority regions.

### Malaria control network

**Fig. 2:**
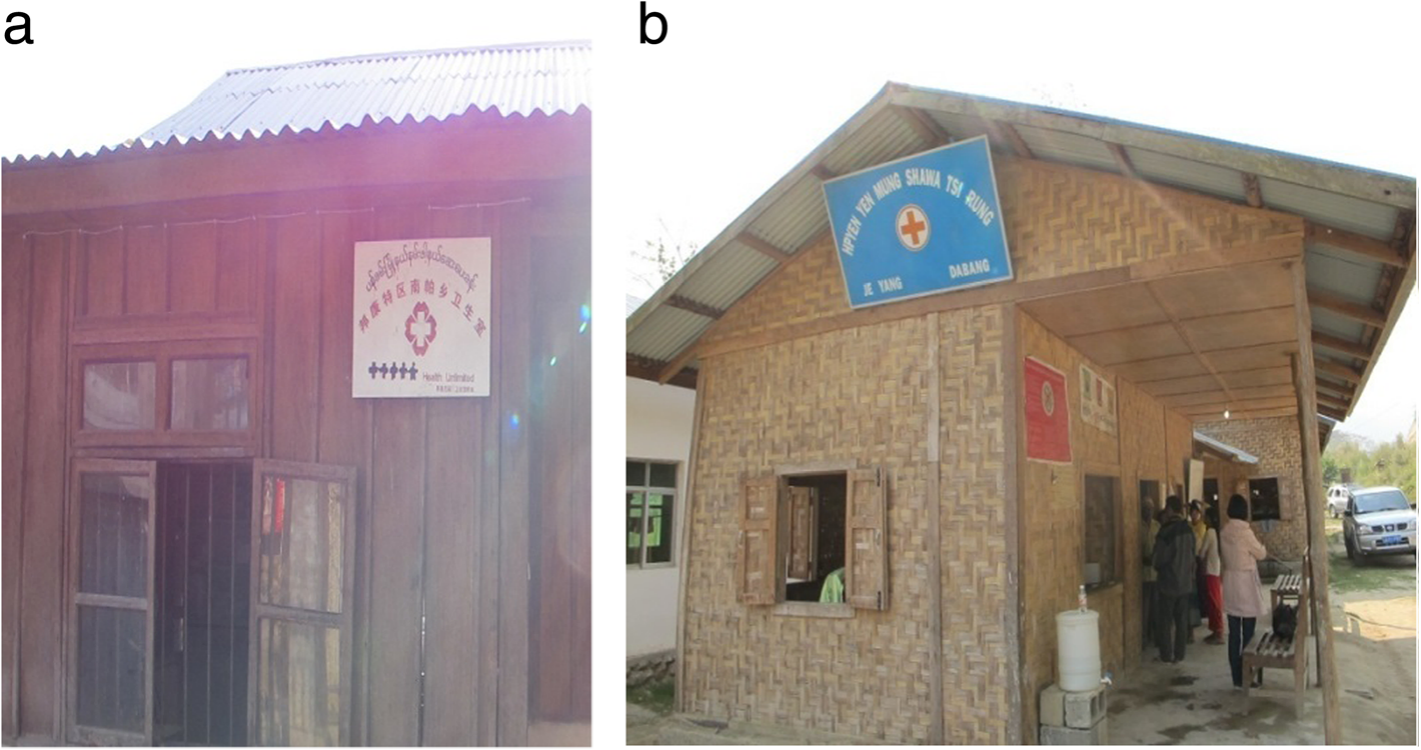
Malaria stations in these regions (**a**: a station in WA; **b**: a station in KSR2)

The malaria control network was gradually formed by the GFATM project. From 2007 to 2011, malaria control activities were conducted by malaria stations (Fig. 2) and outreach teams were formed by the GFATM project. The malaria stations were mainly located at large villages (>400 persons) in these regions. The malaria stations were newly built or renovated, materials (i.e., microscopes, rapid diagnostic tests (RDTs), anti-malaria medicine) were regularly supplied, and one or two recruited doctors were trained to provide malaria services in each malaria station. Malaria outreach services were provided by outreach teams for residents living in remote villages. These outreach teams consisted of qualified and experienced medical doctors, expert microscopists and malaria workers. Since 2012, village malaria workers (VMWs), mobile malaria workers (MMWs) and private clinician physicians were trained to participate in malaria control activities, including diagnosis, treatment. Outreach teams were primarily responsible for malaria outbreak responses, health education, supervision and technical guidance for doctors at malaria stations, VMWs, and MMWs. In 2014, there were 93 malaria stations and 11 outreach teams responsible for malaria control. A total of 56 private clinics and 395 VMWs/MMWs had participated in a variety of malaria control activities.

The malaria information system consists of the malaria information report system and database generated from annual malaria surveys. Malaria information, including diagnosis and treatment, was reported to the outreach teams monthly by malaria stations, VMWs and MMWs. Malaria information was then checked and reported to the HPA by outreach teams and finally reported to the Shan State or Kachin State authorities. The online malaria information database is continuing to expand.

### Interventions

Multiple malaria interventions, including malaria diagnosis, treatment, LLIN distribution and health education, were conducted to improve accessibility and the quality of malaria control services for local residents in the areas covered by the GFATM project.

First, blood samples from febrile patients were analysed via microscopy at malaria stations. Due to the long procurement process of RDTs, there was occasionally a shortage of RDTs at the early stages of the project. Blood samples were also used by outreach teams, and a rigorous quality control system for blood microscopy was established. The microscopists at malaria stations were retrained annually, and 20 % of negative slides and 100 % of positive slides were re-checked by expert microscopists from outreach teams in malaria station laboratories. RDTs were the primary diagnostic tool used by outreach teams and were also distributed to trained VMWs and MMWs.

Second, free malaria treatment was offered at the project cooperation sites (malaria stations, private clinics, VMWs, MMWs and outreach teams). Artemisinin-based combination therapy (ACT) was provided for uncomplicated malaria with *Plasmodium falciparum* and chloroquine plus primaquine (CQ+PQ) therapy was provided for patients with *Plasmodium vivax*. Severe cases of malaria were treated using injectable Artemether, also free of charge. G6PD deficiency tests were not yet performed in these areas. Malaria station doctors, VMWs and MMWs monitored patients for hemolytic reactions after administering primaquine. To improve follow-up compliance, doctors, VMWs and MMWs monitored *P. vivax* patients after treatment with primaquine for a period of 8 days.

As of 2013, all suspected malaria cases received a parasitological test and those with *P. falciparum* received ACT+PQ therapy, according to the guidelines of the Myanmar National Malaria Control Program.

Each year, all of the doctors at the program cooperation sites were trained to improve and reinforce their diagnoses and treatments for uncomplicated and severe malaria. The stock of malaria medicines in health facilities (malaria stations and private clinics) was supervised.

Third, LLINs were the main preventative measure for at-risk populations, which were delivered free of charge each year to poor and vulnerable ethnic residents living in the five Myanmar border regions. As one of the BCC activities, the correct techniques for using LLINs were demonstrated and encouraged. Only in KSR2 were a few nets retreated by KO3 from 2010 to 2011.

Fourth, knowledge, self-protection awareness and behaviours for decreasing malaria transmission (e.g., appropriate bed netting) were instilled in the local residents by outreach teams and trained health education instructors. Many health education activities, such as educational films, performances, poster displays and the distribution of informational leaflets took place in villages and communities, with the participation of the target ethnic minorities. Printed materials focused on only the most crucial knowledge for malaria prevention and were made to be appropriate to local culture and language. Meanwhile, health education columns on malaria were built in villages.

### Malaria surveys

Annual malaria indicator surveys were carried out to evaluate the malaria burden and impact of malaria control activities. Multistage cluster sampling was used to select sample populations, expecting that Probability-Proportional-to-Size Sampling (PPS) was applied in KSR2 in 2014, as most KSR2 residents gathered in refugee camps.

In 2008, baseline surveys were conducted on 5 585 residents and 1 618 households [13]. From 2009 to 2011, all of the villages in each special region were divided into three tiers representing high, middle and low malaria incidence (based on malaria incidence reports from the prior year). Three villages were selected randomly in each tier, and nine villages were selected for each region. A total of 100 residents and 100 households were respectively selected from each village. In general, 100 examined residents belonged to approximately 20–30 households among the 100 households interviewed. Blood samples from residents were examined by microscopy, and households were interviewed about LLIN coverage and usage.

The survey method was revised in 2012. Thirty villages were selected from each region, and 30 residents and ten households were selected from each village. A total of at least 900 residents and 300 households were selected from each region.

A thick blood slide and a thin blood slide were made for each resident. Blood slides were stained with Giemsa Staining Solution and analysed under a microscope. During the survey, all blood slides were re-examined by expert microscopists from outreach teams on the same day. If the re-examination result was below 90 %, all blood slides from the village would be re-examined again.

Meanwhile, a brief survey on health education was conducted in 2013. A total of 5 714 residents over 14 years of age were interviewed with five simple but important malaria questions related to symptoms, transmission, treatment and prevention.

### Data analysis

Databases were generated and analysed using Epidata3.1 and Excel software. Descriptive statistics were evaluated using Microsoft Excel, and 95 % confidence intervals (95 % *CI*s) were determined.

## Results

### Malaria incidence

**Fig. 3:**
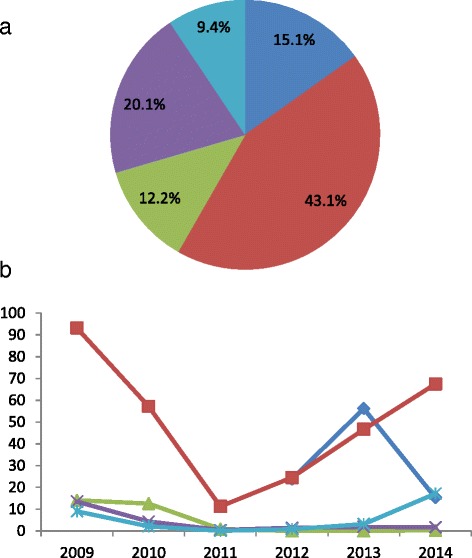
Malaria incidences in the five regions from 2009 to 2014 (KSR1 from 2012 to 2014): (**a**) Proportion of total number of malaria cases by regions; (**b**) Malaria incidence (cases per thousand per year) for each region. (*Blue*: KSR1; *Red*: KSR2; *Green*: KOK; *Purple*: WA; *Cyan*: SR4)

Through passive and active case detection and prevalence surveys, a total of 28 361 malaria cases were detected, treated, and reported from 2009 to 2014. In KSR2, 43.1 % of cases occurred, and 20.1 % occurred in WA, 15.1 % in KSR1, 12.2 % in KOK, and 9.4 % in SR4 (Fig. 3). Malaria cases declined from 10 449 in 2009 to 4 580 in 2014. The annual incidence declined from 19.1 per thousand in 2009 to 8.7 per thousand in 2014. The malaria incidence in KSR2 was always highest among the five regions and the malaria incidences of KOK and WA decreased sharply to 0.3 and 1.7 per thousand by 2014 (Fig. 3). These malaria cases occurred among local residents. Migrants were primarily from China, and they received malaria services upon their return to China or through the purchase of anti-malarial drugs from private venders.

**Fig. 4:**
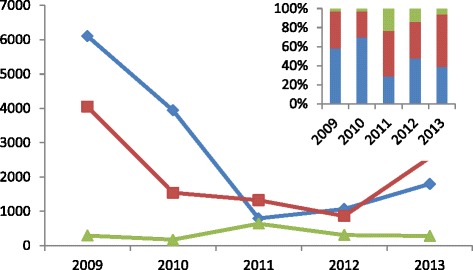
Reported malaria cases in 2009–2013 by plasmodium species (*Blue*: *P.f*; *Red*: *P.v*; *Green*: Other)

A total of 13 706 (53.2 %) *P. falciparum* malaria cases, 10 340 (8.6 %) *P. vivax* malaria cases and 1 700 (6.6 %) other malaria cases were reported from 2009 to 2013. Cases of *P. vivax* and *P. falciparum* declined, and the proportion of *P. vivax* vs. *P. falciparum* cases varied from 0.7:1 in 2009 to 1.4:1 in 2013 (Fig. 4).

**Fig. 5:**
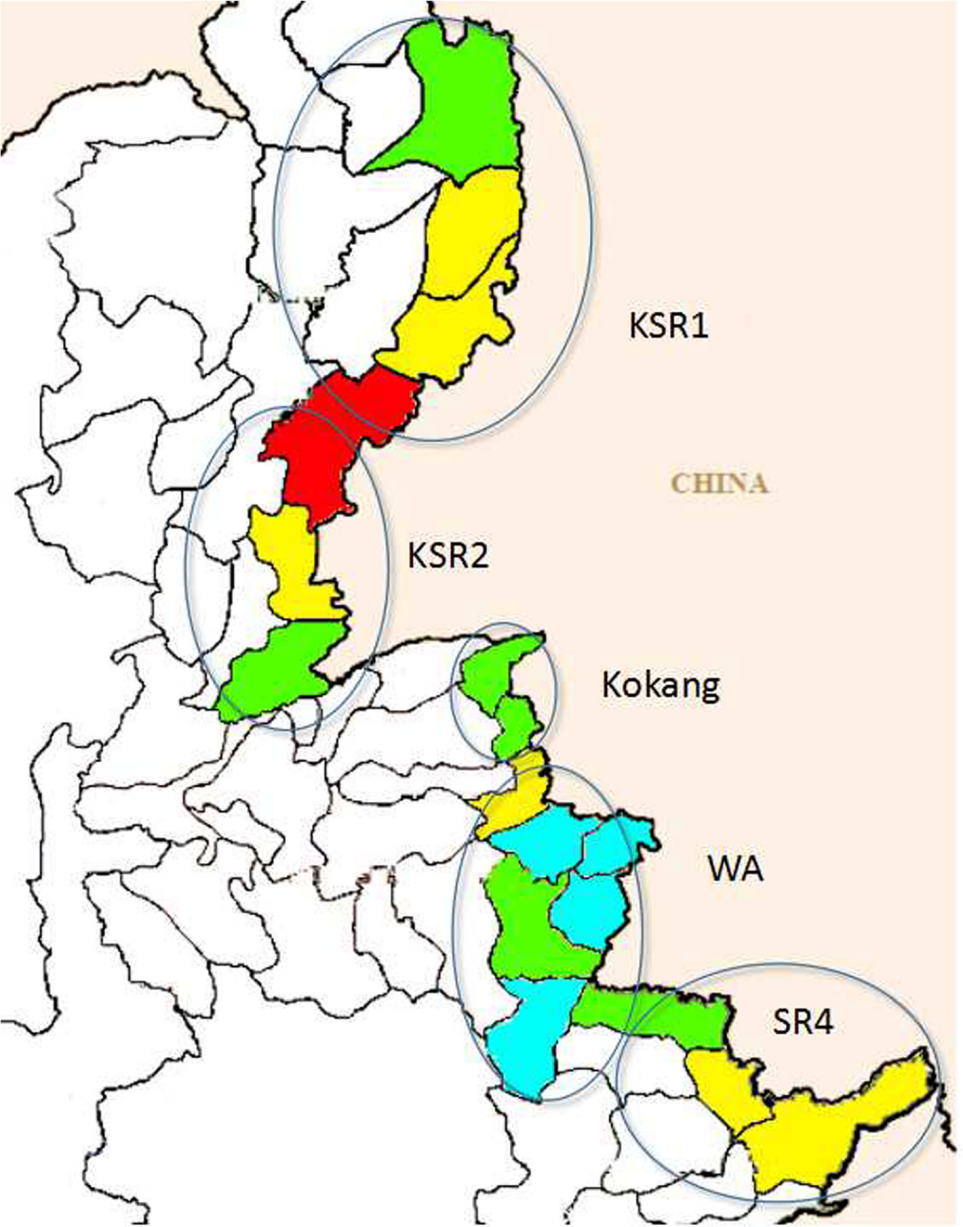
Malaria map in five special regionsin 2014, classified by malaria incidence at township level (*Red*: >50; *Orange*: 50–30; *Yellow*: 30–10; *Green*: 10–1; *Cyan*: <1)

In 2014, a malaria map of these five special regions was made based on malaria incidence at the township level (Fig. 5). Among the 17 townships, the malaria incidence in the Waingmaw Township in KSR2 was highest (more than 50/1 000), and the malaria incidence in the Matman Township in WA was the lowest (no cases occurred in 2014).

### Malaria prevalence

The results of the annual malaria prevalence survey are shown in Table 1. Malaria prevalence decreased from 13.6 % (95 % *CI*: 12.7–14.5 %, 761/5 585) in 2008 to 2.8 % (95 % *CI*: 2.3–3.3 %, 113/4 068) in 2011 in four regions and from 2.2 % (95 % *CI*: 1.7–2.6 %, 98/4 561) in 2012 to 0.4 % (95 % *CI*: 0.2–0.6 %, 20/4 615) in 2014 in five regions. The prevalence of malaria in KOK and WA were lower than that of the other regions (KSR2, SR4 and KSR1) in 2014.

**Table 1:** Prevalence of malaria infection during surveys in regions (%, 95 % *CI*)

Region	2008	2009	2010	2011	2012	2013	2014
KSR1	NA	NA	NA	NA	5.1 (3.7–6.5)	4.7 (3.3–6.1)	0.2 (0–0.5)
KSR2	17.1 (15.0–19.1)	13.3 (11.1–15.6)	13.3 (11.1–15.6)	2.8 (1.9–3.7)	1.1 (0.5–1.7)	1.3 (0.6–2.1)	1.0 (0.3–1.7)
KOK	10.6 (9.0–12.3)	7.9 (6.1–9.7)	6.6 (4.9–8.2)	5.0 (3.6–6.4)	1.9 (0.9–3.0)	0.0	0.0
WA	17.0 (15.1–18.9)	8.9 (7.0–10.7)	8.2 (6.4–10.0)	1.7 (0.8–2.5)	1.6 (0.8–2.4)	0.0	0.0
SR4	9.7 (8.1–11.3)	9.7 (7.7–11.6)	5.9 (4.4–7.4)	1.5 (0.7–2.3)	1.3 (0.6–2.1)	1.7 (0.8–2.5)	1.0 (0.4–1.7)
Total	13.6 (12.7–14.5)	9.9 (9.0–10.9)	8.5 (7.6–9.4)	2.8 (2.3–3.3)	2.2 (1.7–2.6)	1.6 (1.2–1.9)	0.4 (0.2–0.6)

### Interventions

**Fig. 6:**
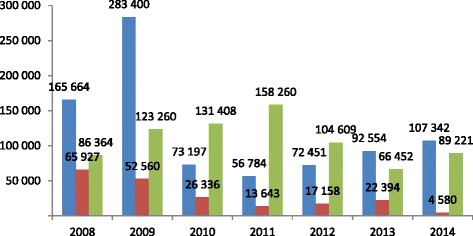
Febrile patient diagnosis, malaria cases (including confirmed cases and suspected cases before 2014), and LLINs distribution in these regions during 2008 to 2014 (*Blue*: Febrile patients through diagnosis test; *Red*: Malaria cases treated; *Green*: LLINs distributed)

Febrile patients (851 393) were detected, and 202 598 malaria cases (including confirmed cases and suspected cases) were treated and 759 574 LLINs were delivered to at-risk populations (Fig. 6).

### Household ITNs/LLINs and Malaria education survey

LLINs (759 574) were delivered free of charge to at-risk populations in the Myanmar program area. Of households in 2012, 73.9 % were observed to have at least one ITN/LLIN, which was a 45.6 % increase compared with the baseline data (28.3 % in 2008). However, the percentage of households, children less than 5 years of age, and pregnant woman who slept under LLINs the night prior to their visit was 41.7, 50.7, and 50.3 %, respectively (Table 2).

**Table 2:** LLINs indicator survey during 2008 to 2014

Indicator	2008	2009	2010	2011	2012	2013	2014
LLINs own-ships	28.3 % (26.1–30.5 %) (458/1 618)	NA	83.0 % (81.8–84.3 %) (2 922/3520)	74.0 % (72.6–75.5 %) (2 713/3 664)	73.9 % (72.4–75.5 %) (2 311/3 126)	NA	NA
Percentage of people who slept under LLIN last night	NA	NA	NA	NA	NA	57.3 % (55.9–58.7 %) (2 638/4 604)	41.7 % (40.9–42.6 %) (5 169/12 383)
Percentage of pregnant woman who slept under LLIN last night	NA	NA	NA	NA	NA	77.5 % (68.9–86.2 %) (69/89)	50.3 % (43.1–57.4 %) (94/187)
Percentage of children under 5 years who slept under LLIN last nigh	NA	NA	47.7 % (46.0–49.3 %) (1 722/3 613)	52.0 % (50.2–53.8 %) (1 585/3 047)	56.9 % (54.9–58.9 %) (1 361/2 392)	72.8 % (69.5–76.0 %) (529/727)	50.7 % (49.0–52.5 %) (1 595/3 145)

Among the 4 576 residents (over the age of 14 years) that visited at 98 villages in 2013, 68.4 % of the residents could correctly answer three or more out of the five malaria questions, while 15.9 % of the residents could not correctly answer any of the five questions.

## Discussion

With increasing malaria control activities supported by international funding, such as the GFATM, there have been great achievements in malaria prevention and treatment in the five ethnic minority regions. Malaria prevalence (13.6 % in 2008; 0.4 % in 2014), annual malaria incidence (19.1 per thousand per year in 2009; 8.7 in 2014) and the overall number of malaria cases (10 449 in 2009; 4 580 in 2014) have decreased dramatically. Similar to the findings reported in the case study from the Thai-Myanmar border areas [8], the present project demonstrates that an integrated malaria control strategy is suitable for improving the quality of malaria control and healthcare accessibility for residents in the border areas of northern Myanmar. The local residents received more diagnostic, treatment, and prevention services from malaria sites established by international NGOs. Furthermore, this project facilitated the expansion of malaria control networks, formed as a local health system, to other communities. In 2014, there were 149 malaria diagnosis sites (93 malaria stations, and 56 private clinics), and 395 VMWs/MMWs conducted malaria services at the community level, a 153 % increase in malaria facilities compared with data in 2007.

Control measures should also account for the differences in the transmission levels. Malaria transmission in KSR2, KSR1, and SR4 is still high, and malaria interventions should cover all of the communities in these regions to decrease their malaria burden. In WA and KOK, the malaria transmission levels are lower. Malaria surveillance should be initiated in these two regions to detect individual malaria cases and assess potential risks of malaria transmission. At low transmission levels, microscopy-based malaria diagnosis is not able to detect malaria cases as well due to low parasitism [14]. It would be feasible to establish one or two central diagnostic laboratories to further improve the malaria diagnosis capabilities and employ PCR methods for malaria surveillance [15, 16].

In addition, *P. falciparum* can be cured with ACT+PQ, but radical cure of *P. vivax* requires a longer treatment with PQ to remove hypnozoites. Since PQ isn’t being given at the 7–14 days, there will be many *P. vivax* relapses; in fact, probably most of the *P. vivax* cases reported here are not new cases. This means that if people are receiving early diagnosis and treatment, the major effect will be in *P. falciparum* cases and subsequently the total proportion of malaria cases should shift toward *P. vivax* being the most prevalent.

In the regions analysed in the present study, malaria transmission was unstable during the project period, and epidemic data were difficult to obtain. A new malaria case report system can be introduced to these regions employing data collection with mobile phones, tablets, and other online devices [17, 18]. Epidemic analyses should be regularly conducted, and sentinel sites should be established to control potential epidemics and understand their patterns and trends. Detailed case information should be collected, reported and analysed regularly.

The percentage of households owning at least one LLIN in 2012 was 73.9 %, which was lower than the target of 80 %. It is essential that LLINs be scaled up for use in all villages and damaged LLINs be replaced [19] with the cooperation of local officials and health workers. Nearly half of the residents in these ethnic minority regions do not sleep under LLINs, signifying a need for more health education about LLIN utilization. Only 68.4 % of residents had some knowledge of the causes, symptoms, treatments and prevention of malaria, but 15.9 % residents had no knowledge. BCC/IEC interventions should be prioritized in these regions [20].

Malaria control was almost completely dependent on international funding and international NGOs in these five regions. Approximately 2 million USD from the GFATM was disbursed for the provision of anti-malarial drugs, RDTs, and LLINs in these areas. Most malaria services were carried out by NGOs under support of the GFATM funds. If international funds are removed, malaria services may be difficult to maintain. For example, the funds were suspended by the GFATM for approximately 6 months during the malaria peak season in 2011–2012. During this period, funds for project logistics were not disbursed or allocated in timely a manner. With a lack of subsidies, some health workers had to leave, which led to the untimely detection and treatment of malaria cases. The international community should invest more funds to these ethnic minority regions for malaria control. International NGOs play an important role in malaria control networks, which serve as a local CDC through implementation of malaria control projects. The government needs to provide a more aggressive political and financial commitment to malaria control strategies.

Regional conflicts often destroy physical infrastructure, collapse health systems and exacerbate the malaria burden [21, 22]. In conflict areas, such as KSR2 and KOK, the malaria activities were carried out continually by health workers, even in the setting of physical danger. Though malaria services were provided in a timely fashion to any refugees, regional conflicts had an overall negative impact on malaria control, reducing the accessibility and quality of malaria control services. Some villages could not be entered by malaria workers, and many villagers who were at risk could not be covered by malaria services. For example, the malaria incidence in KSR2 rebounded from 11.3 per thousand in 2011 to 67.5 per thousand in 2014 after a 2011–2012 conflict. Emergency cooperation systems should be established, and malaria control measures and commodities for emergency response for refugees should be enhanced in KSR2 and KOK.

Cross-border migrants were at risk in the border area and were difficult to cover by malaria services [23–25]. Malaria cases from these ethnic minority regions had been the main source of cases in the Yunnan province of China [26, 27]. A cross-border cooperation mechanism was established in these regions. Malaria information was exchanged regularly between border counties of Yunnan in China and the special regions of Myanmar. Technical assistance was provided to the special regions of Myanmar form neighbouring counties of Yunnan, which improved the technical capacity in these regions. The cross-border cooperation mechanism between Myanmar and China should be maintained and included in the GMS malaria control regulatory board. The sharing of malaria epidemiological data should be a primary concern, and joint malaria control activities for cross border migrations can also be performed. Special activities need to be carried out to improve the accessibility of malaria services for cross-border migrants. There have been some recent political changes in Myanmar, and the central and local governments all support the malaria control activities implemented by international NGOs.

Meanwhile, parasite resistance to anti-malarial drugs remains an obstacle to malaria control and eradication [28]. In Southeast Asia, resistance to the artemisinin family of drugs has been observed [29], which was shown to be a result of mutations in the K13 gene in parasite populations. Indeed, mutations in the K13 gene have been observed in parasite populations near the China-Myanmar border [30, 31]. Surveillance for parasite resistance in the five ethnic minority regions should be initiated immediately.

During the period of 2007 to 2014, the following lessons were learned on promoting malaria control in these regions: malaria control networks from the village to the regional level should be implemented; sustainable funds from the GFATM and appropriate project-implementing agencies (e.g., HPA) are required for project success; cross-border cooperation mechanisms between the border counties of Yunnan and the special regions of Myanmar should be established. Myanmar put forth ambitious goals for achieving malaria pre-elimination by 2020 and elimination by 2030. In these ethnic minority regions, there are many potential challenges underlying this 2020 pre-elimination goal: the sustainability of funding mainly relies on the international community; sustainable malaria control networks and cross-border cooperation mechanisms; lack of professional and technical support in detection methods, malaria control in cross-border migrants, parasite resistance to anti-malarial drugs, surveillance, and so on; and unpredictable political changes and conflicts.

There are several limitations to the present study. First, the malaria prevalence in the five regions of Myanmar may be underestimated by microscopic tests, as a PCR method is more sensitive than microscopy in detecting malaria infections [32]. In western Cambodia, regions in the Thailand–Myanmar border and southwest Vietnam, light microscopy and RDTs identified only one-quarter of all parasitemia participants [33, 34]. A PCR method should be used together with a microscopic test to collect data for a prevalence survey. Second, G6PD deficiencies need to be monitored, and chloroquine/primaquine used for 14 days can be radically cure vivax malaria. A previous study reports that the prevalence of G6PD deficiency is nearly 30 % in the China-Myanmar border area [35]. The long treatment course for primaquine usually results in poor patient compliance and a low treatment efficacy. In these regions, patient compliance for primaquine should be investigated, and patients should be treated with primaquine under medical supervision by doctors, VMWs or MMWs. Health education questionnaires conducted in 2013 were very simple. A detailed survey on health education needs to be conducted and analysed according to the individual questions, place, and so on to design targeted BCC activities [36]. Meanwhile, malaria cases were detected through three methods: 1) passive detection in malaria stations; 2) active detection by outreach teams; and 3) symptomatic cases detected through prevalence surveys. Over the course of this project, case reports did not collect information, such as clinical characteristics or the presence of fever and other symptoms. Furthermore, there are symptomatic carriers and asymptomatic carriers of malaria, and the accuracy of data on the rate of symptomatic carriers was not obtained from these surveys, as the questionnaires did not request symptom information. In the future, we will improve case reports to better document the clinical details of malaria cases and collect symptom information with patient questionnaires.

## Conclusion

From 2007 to 2014, there has been great success in improving malaria control in these five ethnic minority regions. The malaria burdens were decreased, especially in KOK and WA. Maintaining a sustainable malaria control network may be a long-term process in these regions due to regional conflicts and the lack of malaria funds, technology, and health workers. More technical, informational and scientific support from the international community should be offered to these ethnic minority regions to ensure the longevity of these recent successes.

## Additional file

Additional file 1: Multilingual abstract in the five official working languages of the United Nations. (PDF 716 kb)

## Abbreviations

ITNs: Insecticide treated nets
KOK: Kokang Special region in Shan state
KSR1: Special region 1^st^ in Kachin state
KSR2: Special region 2^nd^ in Kachin state
LLINs: Long lasting insecticide-treated nets
RDT: Rapid diagnosis test
SR4: Special region 4^th^ in Shan State
WA: Special region 2^nd^ in Shan State
