# Supplementary material for: Lessons on malaria control in the ethnic minority regions in Northern Myanmar along the China border, 2007–2014
Source: Infect Dis Poverty. 2016 Oct 6;5:95. doi: 10.1186/s40249-016-0191-0 (PMC5053039; doi:10.1186/s40249-016-0191-0)
Supplement: Additional file 1: — Multilingual abstract in the five official working languages of the United Nations. (PDF 716 kb) [file 40249_2016_191_MOESM1_ESM.pdf]

Translation of the abstract into the five official working languages of the United Nations

الدروس المستفادة من مكافحة الملاريا في مناطق الأقليات العرقية في شمال ميانمار على طول الحدود بين الصين، 2007-2014

رو بو وانغ، وشياو تشينغ دونغ، وتشى هوى شيا، وتاو تساى، وتشينغ فنغ تشانغ، وياو تشانغ، ويانغ هوي تيان، وشياو يينغ صن، وغوانغ يون تشانغ، وتشينغ-بو لى، وشياو يو شو، وجيا يين لى وجون تشانغ

تلخيص

معلومات عامة: بالنسبة للعديد من الدول التي تتوطن فيها الملاريا، يزيد عبء الملاريا في المناطق الحدودية. ففي مناطق الأقليات العرقية على طول الحدود بين ميانمار والصين، حيث يحصل السكان الفقراء على نذر قليل من الرعاية الطبية للتشخيص والعلاج، وكما تتفشى الملاريا بشكل كبير في هذه المناطق. ومنذ عام 2007، وفي ظل الدعم من الصندوق العالمي لمكافحة الإيدز والسل والملاريا، جرى تقديم مشروع مكافحة الملاريا للحد من عبء الملاريا في عدة من المناطق التي تقطن بها الأقليات العرقية.

الطرق: أنشئت شبكة مكافحة الملاريا خلال الفترة 2007-2014. وقد أجريت تدخلات متعددة للملاريا، بما في ذلك التشخيص، والعلاج، وتوزيع الناموسيات والتثقيف الصحي، وتحسين إمكانية الوصول ونوعية الخدمات برامج مكافحة الملاريا للسكان المحليين. كما أجريت مسوحات قطاعية سنوية لتقييم تغطية التدخل ومؤشرات انتقال الملاريا.

النتائج: بلغت معدلات الإصابة بمرض الملاريا في مناطق الأقليات العرقية حيث تم إنشاء شبكة لمكافحة الملاريا سنويًا (19.1 في الألف سنويًا، في عام 2009؛ 8.7، في عام 2014)، وانتشار الملاريا (13.6٪ في عام 2008، بنسبة 0.43٪ في عام 2014) وانخفضت بشكل كبير خلال الأعوام الخمس - الست الماضية. وقد جرى توقيع الكشف على إجمالي 851 393 مريضًا بالحمى، كما عولجت 202 598 حالة مصابة بالملاريا (بما في ذلك الحالات المؤكدة والحالات المشتبه فيها)، كذلك تسليم 759574 خطوط للسكان المعرضين للخطر. وبلغت نسبة الأسر التي حصلت على قطعة واحدة على الأقل الناموسيات في عام 2012 73.9٪ (مقابل 28.3٪ في عام 2008)، وينام 50.7٪ من الأطفال ممن هم دون 5 سنوات ونسبة 50.3٪ من النساء الحوامل تحت الناموسيات الليل قبل زيارتهم. إضافة إلى ذلك، تحسنت عملية التعريف بالملاريا لدى 68.4٪ من السكان.

الاستنتاجات: جرى إحراز نجاحًا كبيرًا في تحسين مكافحة الملاريا في هذه المناطق 2007-2014. انخفضت أعباء الملاريا، خاصة في كوك واشنطن. قد تكون الصيانة المستمرة لشبكات مكافحة الملاريا المستدامة في هذه المناطق عملية طويلة الأجل، وذلك نظرًا للصراعات الإقليمية وعدم توافر الأموال والتكنولوجيا، والعاملين في مجال الصحة. وعلاوة على ذلك، ينبغي تقديم المعلومات والدعم العلمي من جانب المجتمع الدولي لهذه المناطق ذات الأقليات العرقية لدعم الإنجازات التي تحققت مؤخرًا.

Translated from English version into Arabic by Fathia Sobhi, throug

TRANSLATORS
WITHOUT BORDERS

中缅边境地区缅甸北部少数民族地区 2007-2014 年疟疾控制

Ru-Bo Wang, Jia-Qiang Dong, Zhi-Gui Xia, Tao Cai, Qing-Feng Zhang, Yao Zhang, Yang-Hui Tian, Xiao-Ying Sun, Guang-Yun Zhang, Qing-Pu Li, Xiao-Yu Xu, Jia-Yin Li, Jun Zhang

摘要

引言：许多疟疾流行国家的边境地区疟疾负担更高。在中缅边境少数民族地区，由于居民难以获得疟疾诊断服务，常常发生疟疾爆发。2007 年以来，在全球抗击艾滋病、结核病和疟疾基金的支持下，疟疾控制项目被引入这些地区，以降低当地的疟疾负担。

方法：2007-2014 年期间，通过建立疟疾控制网络，开展诊断、治疗、长效蚊帐发放以及健康教育等综合性疟疾干预措施，以提高这些地区疟疾诊断治疗服务的可及性和服务质量。通过开展年度横断面调查，评估干预措施覆盖率和指标完成情况。

结果：在中缅边境缅甸一侧的少数民族地区，建立了疟疾控制网络。在过去的 5-6 年间，疟疾发病率从 2009 年的 19.1/1000 下降至 2014 年的 8.7/1000；疟原虫带虫率从 2008 年的 13.6%下降至 2014 年的 0.43%。累计检测发热病人 851393 例，治疗疟疾病例（包括确诊和疑似病例）202598 例，发放长效蚊帐 759574 例。2012 年调查显示，73.9%的家庭拥有至少一顶长效蚊帐或药浸蚊帐（2008 年仅为 28.3%），50.7%的 5 岁以下儿童和 50.3% 的孕妇睡眠时使用长效蚊帐。此外，68.4%的居民具有疟疾防治知识。

结论：2007-2014 年间，这些少数民族地区疟疾控制取得了巨大进步，降低了当地的疟疾负担，特别是在果敢地区和佤邦。由于地区冲突以及防治资金、技术、人员的缺乏，疟疾控制网络的持续维护将是一个长期的过程。此外，国际社会应提供更多的技术支持，以维持近年来取得的成绩。

Translated from English version into Chinese by Ru-Bo Wang

CHINESE CENTER FOR DISEASE CONTROL AND PREVENTION
NATIONAL INSTITUTE OF PARASITIC DISEASES

Leçons relatives à la lutte contre le paludisme dans les régions des minorités ethniques dans le nord du Myanmar à la frontière avec la Chine, 2007-2014

Ru-Bo Wang, Jia-Qiang Dong, Zhi-Gui Xia, Tao Cai, Qing-Feng Zhang, Yao Zhang, Yang-Hui Tian, Xiao-Ying Sun, Guang-Yun Zhang, Qing-Pu Li, Xiao-Yu Xu, Jia-Yin Li, Jun Zhang

Résumé

Contexte : le fardeau du paludisme est plus élevé dans les régions frontalières dans de nombreux pays où cette maladie est endémique. Dans les régions des minorités ethniques le long de la frontière entre le Myanmar et la Chine, les résidents ne disposent que d'un accès insuffisant aux soins médicaux de diagnostic et de traitement et de nombreuses épidémies de paludisme se sont produites dans ces zones. Depuis 2007, un projet de lutte contre le paludisme a été lancé avec l'aide du Fonds mondial de lutte contre le sida, la tuberculose et le paludisme (FMSTP) afin de réduire le fardeau du paludisme dans plusieurs régions des minorités ethniques.

Méthodes : un réseau de lutte contre le paludisme a été établi au cours de la période 2007-2014.

Des interventions multiples de lutte contre le paludisme incluant le diagnostic, le traitement, la distribution de moustiquaires imprégnées d'insecticide de longue durée et des sessions de sensibilisation à la santé ont eu lieu afin d'améliorer l'accessibilité et la qualité des services de lutte contre le paludisme pour les résidents locaux. Des enquêtes transversales annuelles ont été réalisées afin d'évaluer la couverture de l'intervention ainsi que les indicateurs de transmission du paludisme.
Résultats : dans les régions des minorités ethniques où un réseau de lutte contre le paludisme a été établi, l'incidence du paludisme (19,1 par millier par an en 2009 ; 8,7 en 2014) et sa prévalence annuelles (13,6 % en 2008 ; 0,43 % en 2014) ont nettement diminué au cours des 5 à 6 années précédentes. Au total, 851 393 patients fébriles ont été détectés, 200 598 cas de paludisme (réunissant les cas confirmés et soupçonnés) ont été traités et 759 574 moustiquaires imprégnées d'insecticide de longue durée ont été distribuées aux populations à risque. En 2012, 73,9 % des ménages disposaient d'au moins une moustiquaire imprégnée d'insecticide de longue durée (vs 28,3 % en 2008), 50,7 % des enfants de moins de 5 ans et 50,3 5 des femmes enceintes avaient dormi sous des moustiquaires imprégnées d'insecticide de longue durée la veille de la visite. De plus, les connaissances relatives au paludisme ont été améliorées chez 68,4 % des résidents.
Conclusions : la lutte contre le paludisme a été nettement améliorée dans ces régions de 2007 à 2014. Le fardeau du paludisme a diminué, notamment dans les régions KOK et WA. La maintenance continue de réseaux de lutte durable contre le paludisme dans ces régions peut représenter un processus à long terme en raison des conflits régionaux et de l'absence de fonds, de technologies et d'agents de santé. De plus, des informations et une assistance scientifique de la part de la communauté internationale devrait être offertes à ces régions des minorités ethniques afin de maintenir les résultats récents.

Translated from English version into French by eric ragu, through

TRANSLATORS
WITHOUT BORDERS

Уроки борьбы с малярией в районах проживания этнических меньшинств в Северной Мьянме вдоль китайской границы, 2007-2014 гг.

Жу-Бо Ван (Ru-Bo Wang), Цзя-Цян Дун (Jia-Qiang Dong), Чжи-Гуй Ся (Zhi-Gui Xia), Тао Цай (Tao Cai), Цин-Фэн Чжан (Qing-Feng Zhang), Яо Чжан (Yao Zhang), Ян-Хуэй Тянь (Yang-Hui Tian), Сяо-Ин Сунь (Xiao-Ying Sun), Гуан-Юнь Чжан (Guang-Yun Zhang), Цин-Пу Ли (Qing-Pu Li), Сяо-Юй Сюй (Xiao-Yu Xu), Цзя-Инь Ли (Jia-Yin Li), Цзюнь Чжан (Jun Zhang)

Реферат

Предыстория
Во многих странах, где малярия носит эндемический характер, отмечаются более высокие масштабы заболеваемости малярией в пограничных районах. В районах проживания этнических меньшинств в Северной Мьянме вдоль границы между Мьянмой и Китаем жители не имеют достаточного доступа к медицинскому обслуживанию для диагностики и

лечения, и в таких районах наблюдаются многочисленные вспышки малярии. При поддержке Глобального фонда по борьбе со СПИД, туберкулезом и малярией (GFATM) в 2007 г. для уменьшения бремени малярии в нескольких районах проживания этнических меньшинств учрежден проект по борьбе с малярией.
Методы: В 2007-2014 гг. была создана система борьбы с малярией. Для улучшения доступности и качества услуг по борьбе с малярией для местного населения проводились мероприятия по борьбе с малярией, в том числе, диагностика, лечение, распределение ОИПС (обработанных инсектицидом противомоскитных сеток) и санитарное просвещение. Проводились ежегодные перекрестные обследования для оценки охвата мероприятиями и показателей передачи малярии.
Результаты: За последние 5-6 лет в районах проживания этнических меньшинств, где создана система борьбы с малярией, ежегодное число случаев заболевания малярией (19,1 на тысячу человек в год в 2009 г.; 8,7 в 2014 г.) и показатели распространенности малярии (13,6% в 2008 г.; 0,43% в 2014 г.) значительно снизились. Всего выявлено 851 393 лихорадочных больных, проведено лечение 202 598 случаев малярии (включая подтвержденные и подозрительные случаи) и передано уязвимому населению 759 574 москитных сетки. В 2012 г. 73,9% всех семейств были обеспечены по крайней мере одной ОИПС/ОИСДП (по сравнению с 28,3% в 2008 г.); 50,7% детей в возрасте до 5 лет и 50,3% беременных женщин спали под москитными сетками в ночь перед посещением их жилья. Кроме того, санитарное просвещение улучшилось среди 68,4% жителей.
Выводы: В 2007- 2014 гг. в данных регионах отмечено значительное улучшение борьбы с малярией. Бремя малярии снизилось, особенно в районах Кок и Ва. Дальнейшее сохранение устойчивой системы борьбы с малярией в этих регионах может быть долговременным процессом из-за региональных конфликтов и отсутствия фондов, технологии и медико-санитарных работников. Более того, для сохранения недавних достижений необходимо оказать этим районам проживания этнических меньшинств информационную и научную поддержку международного сообщества.

Translated from English version into Russian by Tatyana Johnson, through

TRANSLATORS
WITHOUT BORDERS

Lecciones sobre el control del paludismo en las zonas fronterizas con China del norte de Myanmar donde residen las minorías étnicas (2007 – 2014)

Ru-Bo Wang, Jia-Qiang Dong, Zhi-Gui Xia, Tao Cai, Qing-Feng Zhang, Yao Zhang, Yang-Hui Tian, Xiao-Ying Sun, Guang-Yun Zhang, Qing-Pu Li, Xiao-Yu Xu, Jia-Yin Li, Jun Zhang

Resumen
Antecedentes: En muchos países donde el paludismo es endémico, la carga de esta enfermedad es más alta en las zonas fronterizas. En las zonas fronterizas entre Myanmar y China donde residen las

minorías étnicas, apenas hay acceso a una asistencia sanitaria para el diagnóstico y tratamiento, y se han dado muchos brotes de paludismo. Desde 2007, con el apoyo del Fondo Mundial de Lucha contra el SIDA, la Tuberculosis y el Paludismo (GFTAM, por sus siglas en inglés), se ha introducido un proyecto de control del paludismo para reducir la carga de esta enfermedad en varias zonas con minorías étnicas.
Métodos: De 2007 a 2014 se creó una red de control del paludismo. Con el objetivo de mejorar el acceso y la calidad de los servicios de control del paludismo entre los residentes de la zona, se llevaron a cabo numerosas intervenciones contra el paludismo, entre ellas el diagnóstico, el tratamiento y la distribución de MTI (mosquiteros tratados con insecticida) de acción prolongada y educación en materia de salud. Cada año se realizaron estudios transversales para evaluar la cobertura de las intervenciones y los indicadores de transmisión del paludismo.
Resultados: En las zonas con minorías étnicas donde se creó la red de control del paludismo, tanto la incidencia anual de paludismo (19,1 ‰ en 2009 y 8.7 ‰ en 2014) como la prevalencia (13,6 % en 2008 y 0,43 % en 2014) descendieron de manera acusada durante los pasados 5 a 6 años. Se detectaron un total de 851 393 pacientes con fiebre, se trataron 202 598 casos de paludismo (tanto casos confirmados como supuestos), y se suministraron 759 574 MTI de acción prolongada a las poblaciones en riesgo. En 2012, en el 73,9 % de los hogares había al menos un MTI o MTI de acción prolongada (frente al 28,3 % en 2008), y el 50,7 % de los niños de menos de 5 años y el 50,3 % de las mujeres embarazadas durmieron protegidos por MTI de acción prolongada durante la noche antes de la visita. Además, el 68,4 % de los habitantes mejoró sus conocimientos sobre el paludismo.
Conclusiones: De 2007 a 2014, el control del paludismo ha mejorado considerablemente en estas regiones. La carga del paludismo ha disminuido, sobre todo en KOK y WA. Debido a los conflictos y a la falta de financiación, tecnología y personal sanitario en estas regiones, es posible que el proceso de mantenimiento continuado de redes sostenibles de control del paludismo sea largo. Además, en estas regiones con minorías étnicas, se debería ofrecer una información y un respaldo científico por parte de la comunidad internacional para que los logros recientes se mantengan.

Translated from English version into Spanish by Mar Jiménez Quesada, through

TRANSLATORS
WITHOUT BORDERS
